# The combination of urinary IL - 6 and renal biometry as useful diagnostic tools to differentiate acute pyelonephritis from lower urinary tract infection

**DOI:** 10.1590/S1677-5538.IBJU.2016.0049

**Published:** 2016

**Authors:** Sherif Azab, Mostafa Zakaria, Mona Raafat, Hadeel Seief

**Affiliations:** 1 Department of Urology, Faculty of Medicine, October 6 University, Cairo, Egypt; 2 Department of Pediatric, Cairo University, Cairo, Egypt; 3 Department of Clinical Pathology, National research Center, Cairo, Egypt; 4 Department of Radiology, University Cairo, Cairo, Egypt

**Keywords:** Ultrasonography, Lower Urinary Tract Symptoms, Interleukin-6, Pyelonephritis

## Abstract

**Objective:**

To evaluate the role of renal ultrasound (RUS) and urinary IL-6 in the differentiation between acute pyelonephritis (APN) and lower urinary tract infection (LUTI).

**Patients and methods:**

This prospective study was carried out at the Pediatric and urology outpatient and inpatient departments of Cairo University Children's Hospital as well as October 6 University Hospital and it included 155 children between one month and fourteen years old with positive culture UTI. Patients were categorized into APN and LUTI based on their clinical features and laboratory parameters. Thirty healthy children, age and sex matched constituted the control group. Children with positive urine cultures were treated with appropriate antibiotics. Before treatment, urinary IL-6 was measured by enzyme immunoassay technique (ELISA), and renal ultrasound (RUS) was done. CRP (C-reactive protein), IL-6 and RUS were repeated on the 14th day of antibiotic treatment to evaluate the changes in their levels in response to treatment.

**Results:**

UIL-6 levels were more significantly higher in patients with APN than in patients with LUTI (24.3±19.3pg/mL for APN vs. 7.3±2.7pg/mL in LUTI (95% CI: 2.6-27.4; p<0.01). Similarly, serum CRP was more significantly higher in patients with APN than in children with LUTI (19.7±9.1μg/mL vs. 5.5±2.3μg/mL (p<0.01). IL-6 levels >20pg/mL and serum CRP >20μg/mL were highly reliable markers of APN. Mean renal volume and mean volume difference between the two kidneys in the APN group were more than that of the LUTI and control groups (P<0.001). Renal volume between 120-130% of normal was the best for differentiating APN from LUTI.

**Conclusions:**

RUS and urinary IL-6 levels have a highly dependable role in the differentiation between APN and LUTI especially in places where other investigations are not available and/ or affordable.

## INTRODUCTION

Urinary tract infections (UTIs) remain one of the most common bacterial infections in infants and children (1). They are important in view of their acute morbidity and long term risk of renal scaring. In UTIs differentiation between acute pyelonephritis (APN) and lower urinary tract infection (LUTI) is recommended because of their therapeutic and prognostic consequences (2).

Traditionally accepted methods for the diagnosis and follow-up of pediatric UTIs need to be periodically assessed and should be discarded if they can be substituted by other methods that are less invasive and more effective. In recent years, the clinical value of routine renal ultrasound (RUS) for young children in whom a first UTI is diagnosed has been questioned because of its limited effect of findings on clinical management (3). However, several investigators documented a significant volume increase with acute infection in one or both kidney(s) of those children having APN (4).

Defense against mucosal infections relies on chemokines that engage inflammatory cells to the mucosa (5). Pro-inflammatory cytokines, particularly interleukin-6 (IL-6), determined in urine (uIL-6) or serum (sIL-6), have been shown to be useful as parametric biological indicators of renal envolvement in UTI (6, 7). IL-6 is secreted by uroepithelial cells in response to bacterial invasion, particularly P-fimbriated Escherichia coli (8). In healthy children, IL-6 is found in only small quantities; however, these levels show a significant increase in patients with renal disease (9–11).

The present study included children with urinary tract infection (UTI), to evaluate the usefulness of measuring uIL-6 levels and RUS in distinguishing APN from LUTI.

## PATIENTS AND METHODS

This prospective study was carried out at the Pediatric and Urology outpatient and inpatient departments of Cairo University Children's Hospital as well as October 6 University Hospital. The study population included children between one month and fourteen years old who were diagnosed with symptomatic culture positive first episode of UTI between March 2009 and March 2012. Children who had been treated with antibacterial agents within seven days before the admission were excluded.

Diagnosis of UTI was performed on the basis of suggestive clinical symptoms and at least one positive urine culture (colony counts >100.000 bacteria/mL). Patients were classified into APN and LUTI based on their clinical features and laboratory parameters. Diagnostic parameters for APN included the presence of all of the following criteria: loin pain, body temperature >38.5°C, total leucocyte count (TLC) ≥12000mm3, erythrocyte sedimentation rate ≥20mm/hour, and leukocyte casts in urinalysis (2). Patients were judged for lower UTI by fulfilling the clinical criteria including pain, frequency, urgency and dysuria, which was mentioned as crying during urination in infants. The laboratory data was suggestive of lower urinary tract infection when TLC was < 12000/mm3 and erythrocyte sedimentation rate <20mm/hour.

Urine analysis, culture and sensitivity were done by collecting morning midstream samples and by suprapubic aspiration in young infants. Finally, one hundred and fifty five children out of two hundred satisfied the study criteria and were enrolled in the study. The control group comprised thirty healthy children, with age and sex matched.

Serum CRP (C-reactive protein) was measured by quantitative enzyme linked immunosorbent assay.

Urinary IL-6 was measured by quantitative sandwich enzyme immunoassay technique (Quantakine ELISA catalog number D6050).

Renal ultrasound (RUS) was done using Siemens Elegra ultrasound machine. Measurement of the kidney was made in the maximum longitudinal and transverse planes, both of which were identified visually. The renal long axis is usually seen opened caudally to the median body plane at an angle of 10°. The transverse section was defined in the kidney hilar region at a right angle to the longitudinal renal axis. The planes were frozen at the screen and the major and minor kidney axes - the bipolar kidney length, width and depths were measured. The absolute volume of the kidney was calculated and compared with the average kidney volumes. The average absolute kidney volume of a normal person of distinct body weight is defined as 100%. Difference between this average volume and the volume of a patient's single kidney were expressed as percentage of the absolute kidney volume.

The mathematical formula used to calculate the absolute kidney volume was:

LXW (D1+D2)/2 X 0.523cm^3^ where L=maximum bipolar length of the kidney, W=maximum width in kidney hilar region, D1=depth in the longitudinal plane, D2=depth in the transverse plane (12).

Children with positive urine cultures were treated with appropriate antibiotics. CRP, IL-6 and RUS were repeated on the 14th day of the antibiotic treatment, to note the changes in these parameters in response to treatment.

### Statistical analysis

Data were statistically described in terms of mean±standard deviation (SD), median and range, or frequencies (number of cases) and percentages when appropriate. Comparison of numerical variables between the study groups was done using Student t test. For comparing categorical data, Chi square (χ2) test was performed. Correlation between various variables was done using Spearman rank correlation equation. p values less than 0.05 were considered statistically significant. The Receiver Operating Characteristic (ROC) Curve was constructed to obtain the most sensitive and specific cutoff for each technique. All statistical calculations were done using computer programs SPSS (Statistical Package for the Social Science; SPSS Inc., Chicago, IL, USA) version 15 for Microsoft Windows.

## RESULTS

Of the total 155 cases with UTI, 70 (45%) were labeled as APN and 85 (55%) as LUTI. The majority of the patients (72%) presented between 4-14 years of age; 80% were females, with female to male ratio 4:1. On urine culture, E. coli was the commonest grown organism: (110 cases: 71%), 70 cases (63%) in LUTI, and 40 cases (37%) in LUTI. Other organisms isolated were Klebsiella in 20 cases (13%), Proteus in 16 cases (10%), Staph. aureus in 7 cases (5%) and others in the remaining 2 cases (1%).

In the current study, uIL-6 levels at diagnosis were significantly higher in patients with APN than in patients with LUTI (24.3±19.3pg/mL for APN vs. 7.3±2.7pg/mL in LUTI (95% CI: 2.6-27.4; p<0.01). Similarly, sCRP was significantly higher in patients with APN than in children with LUTI: 19.7±9.1μg/mL vs. 5.5±2.3μg/mL respectively (p<0.01) (Table-1). Follow-up measurement of sCRP and uIL-6 after 14 days of the antibiotic therapy revealed undetectable levels in both groups of patients. The difference between uIL-6 and sCRP levels at diagnosis and recovery was statistically significant (p<0.05).

**Table 1:** Laboratory data of the study and control groups.

	APN (n = 70)	LUTI (n = 85)	Control (n = 30)	p value*
CRP(µg/mL)	19.7±9.1	5.5±2.3	0.09±0.1	<0.001
uIL-6 (pg/mL)	24.3±19.3	7.3±2.7	3.1±1.2	<0.001
Hemoglobin (gm %)	9.82±2.2	10.12±2.2	11.93±1.4	<0.05
TLC (per mm3)	24600±3200	11284±1160	6020±512	<0.001
ESR (mm/1st hour)	38±2.1	15±2.3	8.4±4.9	<0.001
Urinalysis Albuminuria: n (%)	56 (80%)	17 (20%)	0	<0.001
Pyuria: n (%)	70 (100%)	77 (89%)	0	<0.05
RBC: n (%)	42 (60%)	17 (20%)	0	<0.05
White cell cast: n (%)	70 (100%)	No (0%)	0	<0.001

Table-1 shows comparison of the laboratory parameters in the study and control group.

Using Receiver Operator Characteristic (ROC) Curve, we found that the area below the ROC curves was 0.86 (95% CI: 0.73-0.98), and 0.67 (95% CI: 0.53-0.8) for sCRP, and uIL-6, respectively. Table-2 shows sensitivity, specificity, positive predictive value (PPV), and negative predictive value (NPV) for uIL-6) in the diagnosis of APN.

**Table 2:** Sensitivity, specificity and predictive value of urinary interleukin-6 (uIL-6) in the diagnosis of acute pyelonephritis (APN).

	Sensitivity (%) (95% CI)	Specifcity (%) (95% CI)	Predictive value (%) (95% CI)	
			Positive	Negative
uIL-6>2pg/mL	79.9 (76.9-82.7)	57.2 (51.8-62.9)	65.8 (62.3-70.1)	72.4 (65.8-72.1)
uIL-6>20pg/mL	39.9 (34.9-42.9)	95.1 (92.1-96.2)	88.2 (79.1-90.8)	60.3 (55.3-60.2)

**CI** = Confidence interval

**Figure 1:**
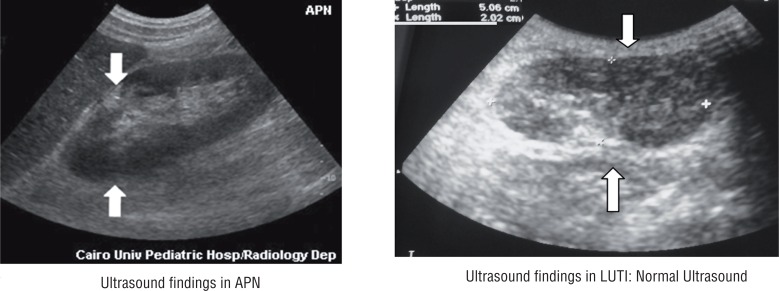
Ultrasound findings in the study cases.

Mean renal volume and mean volume difference between the two kidneys in the APN group was more than the LUTI and control groups (P<0.001) (Table-3). Increase in renal volume was caused by parenchymal thickening and not by an enlarged central echo complex (Figure-1). No focal lesions were detected on US. Renal parenchymal cortical-medullar differentiation was lost in most cases of APN.

**Table 3:** Renal volume assessment by ultrasound in patients with APN and LUTI.

	APN (n: 70)	LUTI (n: 85)	Control (n: 30)	P value
Mean volume of large kidney (%)	188.5±65.6	99.6±12.5	100±20	<0.001
Mean of difference in volume (%)	48.1±9.1	9.9±3.5	8.9±2	<0.001

Table-3 shows the mean renal volume and the mean volume difference between the two kidneys in the study cases.

Table-4 shows sensitivity, specificity, PPV and NPV of renal biometry in the diagnosis of APN.

**Table 4:** Sensitivity, specifcity and predictive values of renal biometry in the diagnosis of APN.

Volume of larger kidney	Sensitivity ratio	Specifcity ratio	Predictive value (%)	
			Positive test	Negative test
>120%	62/70 (89)	76/85 (89)	62/69 (90)	76/85 (89)
>130%	59/70 (84)	82/85 (96)	59/62 (95)	82/93 (88)
>140%	55/70 (79)	85/85 (100)	52/52 (100)	85/102 (83)

**Sensitivity** = probability of a positive finding in a patient with acute pyelonephritis; **Specifcity** = probability of a negative finding in a patient without acute pyelonephritis, that is, with acute lower UTI; **Predictive value of positive test** = percentage of patients with positive test results who have acute pyelonephritis; **Predictive value of negative test** = percentage of patients with negative test results who have acute lower UTI

Kidney asymmetry was diagnosed if the difference in the volume of a kidney pair was more than 20%. In APN, asymmetry was present in 30%; volume difference of more than 40% occurred in 21% of these patients. Mean volume difference was 30.2%. Follow-up assessment of renal volume was done in 20 patients of APN, and showed an average of 60% reduction in volume of the affected kidney (from the first recorded value in the study) after 2 weeks of successful antibiotic therapy.

## DISCUSSION

Our study confirms the usefulness of determining urinary IL-6 to differentiate between APN and LUTI in children and also indicates that the process of recovery from the infection results in normalization of urinary IL-6 levels. This is in agreement with the results of Miguel et al. (13) and Sheu et al. (14). Serum and urinary levels of IL-6 increase during UTI, and its measurement have been suggested to be of value in differentiating between APN and lower UTI (7, 15, 16). However, measurement of urinary IL-6 is less aggressive than determination of serum levels and therefore more acceptable for patients and health professionals.

Moreover, our results show that, based on the specificity and sensitivity of the measurement, in the presence of clinical manifestations indicative of UTI, IL-6 levels >20pg/mL is a highly reliable marker of APN. Comparable findings were observed by Miguel et al. (13) who suggested a level >15pg/mL is an excellent marker of APN and concluded that a value of urinary IL-6 greater than 15pg/mL is 6.33 times more likely to be found in patients with APN than in those with lower UTI.

The efficiency of measuring urinary IL-6 in the diagnosis of UTI was assessed using two cutoff points, one standardized as positive or negative by the reference laboratory, and the other with utmost competence for differentiating APN from lower UTI obtained by creating ROC curves (17).

Although the current study was not designed to investigate the value of uIL-6 as a marker of therapeutic response, our results obviously showed that clinical remission is associated with normalization of uIL-6 levels. Moreover, Tullus et al. (11) in a former study demonstrated that persistent renal scarring, a year after APN, was evident only in children with increased urinary levels of IL-6. We concluded that the follow-up of uIL-6 levels in patients with APN can help us judge the proper duration of antibiotic treatment during the acute phase, and it also might be used as an indicator for the risk of persistent renal damage.

CRP is an acute phase reactant produced in the liver. Data from different studies support that elevated sCRP levels are useful for the discrimination between APN and LUTI in patients with distinct clinical signs of APN (18, 19). In the present study, the mean sCRP level in the APN group was significantly higher than that in LUTI as well as the control groups. The cutoff point for maximum diagnostic efficiency of sCRP in patients with APN was 65mg/L. On the other hand, Miguel et al. (14) suggested a sCRP level of 70mg/L as the most competent level for diagnosis of APN. Our results also confirm the observation of Dinkel et al. (2) who noted that CRP value <20μg/mL was suggestive of LUTI (2). Nevertheless, other studies have noted values of up to 30μg/mL in LUTI (20, 21).

In the current study, we could demonstrate that ultrasound also allows a reasonable level of differentiation between APN and lower UTI. In our study, patients with LUTI showed mean volume of the larger kidney to be 99.6±11.4% of normal; mean volume difference between the two kidneys was 9.9±3.59%. Comparable values were reported by Dinkel et al. (2) who reported 99.7% and 12.3% respectively and Khan et al. (19) who suggested a mean volume of 95.2±15.4% and mean volume difference of 9.0±4.7% in patients with LUTI. These observations confirm that there is no significant alteration in the mean renal volume and mean volume difference in patients with LUTI.

Among patients with APN a significant increase in mean kidney size (188.5±65.6) and mean volume difference (48.1±9.14%) was noted (P<0.001). Nearly 80% of patients with APN had a renal volume exceeding 140% of normal. Similar figures of 175.8% and 30.2% mean renal volume and mean volume difference respectively have been reported by Dinkel et al. (2); 76% of the cases in their study had renal volume exceeding 140%. On the basis of these observations it is evident that the increase in renal size and unilateral renal enlargement are two important features of APN. The sensitivity, specificity and predictive value of renal biometry in the differentiation of acute UUTI from LUTI varied in our population according to whether a volume increase in larger kidney of 120%, 130% or 140% were chosen as the critical values (Table-4). We observed that kidney volume between 120-130% of normal is best for differentiating APN from LUTI, because of the higher sensitivity of this cut-off range compared to those with a value >140% this is in agreement with results of Khan et al. (19). Moreover, in the current study, follow-up of 20 patients with APN, revealed nearly a 60% reduction in the renal volume within 14 days. Similarly, Khan et al. (19) documented nearly a 43.6% reduction in the renal volume within the same follow-up period, and Dinkel et al. (2) demonstrated a 20-45% reduction in the renal volume within 6 days and 60% within 2 weeks of treatment.

Renal ultrasound as an investigation in children with first UTI has been recommended by several studies (22, 23). However, most workers emphasize that US although has high specificity in detecting APN and obstructive uropathy, its sensitivity is only modest especially while detecting vesicoureteral reflux and renal scarring (24). Therefore, it has been recommended that children under 5 years of age with recurrent UTI should be further subjected to micturating cystourethrogram (MCU) and renal dimercaptosuccinate acid (DMSA) scan if their US is normal. In a comparative study of US and DMSA scan in the same patients, it was found that US failed to detect half of the kidneys with scars (25). However, getting a DMSA scan in all patients with UTI is practically not feasible because this test, besides being more costly, it is not available in most centers.

## CONCLUSIONS

Renal US and urinary IL-6 levels have a very important role in the differentiation between APN and LUTI especially in those who were either not clearly diagnosed with acute pyelonephritis or lower urinary tract infection by clinical criteria and routine laboratory investigations and in places where other investigations are not available and / or affordable. Urinary IL-6 level >20pg/mL along with increased renal volume should be taken as highly suggestive indicators of APN. Moreover, serial estimation of these parameters may have prognostic significance as well as an aid in monitoring response to therapy.
